# Geographic patterns of ovarian tissue cryopreservation utilisation across age groups in a nationwide multicentre cohort

**DOI:** 10.1093/hropen/hoag076

**Published:** 2026-08-13

**Authors:** Norah L A Emrich, Rebekka Einenkel, Cara M Färber, Andreas Schallmoser, Nicole Sänger

**Affiliations:** Department of Gynaecological Endocrinology and Reproductive Medicine, University of Bonn, University Hospital Bonn, Bonn, Germany; Department of Gynaecological Endocrinology and Reproductive Medicine, University of Bonn, University Hospital Bonn, Bonn, Germany; Department of Gynaecological Endocrinology and Reproductive Medicine, University of Bonn, University Hospital Bonn, Bonn, Germany; Department of Gynaecological Endocrinology and Reproductive Medicine, University of Bonn, University Hospital Bonn, Bonn, Germany; Department of Gynaecological Endocrinology and Reproductive Medicine, University of Bonn, University Hospital Bonn, Bonn, Germany

**Keywords:** ovarian tissue cryopreservation, fertility preservation, oncofertility, geographic disparities, fertility utilisation, paediatric patients, referral networks, urban–rural differences

## Abstract

**STUDY QUESTION:**

Does geographic proximity to a centralised cryobank reflect age-dependent differences in ovarian tissue cryopreservation (OTC) utilisation?

**SUMMARY ANSWER:**

Paediatric patients resided significantly closer to the cryobank than adolescents and adult women, suggesting age-dependent geographic differences in the utilisation of fertility preservation services.

**WHAT IS KNOWN ALREADY:**

OTC is an established fertility preservation option, and previous studies have primarily focused on return rates of cryopreserved ovarian tissue and subsequent pregnancy and live birth outcomes. However, less is known about utilisation patterns of fertility preservation, particularly for OTC in paediatric oncology, where utilisation may be influenced by factors such as clinician-initiated counselling and local referral pathways.

**STUDY DESIGN, SIZE, DURATION:**

This retrospective nationwide multicentre cohort study included 2,426 patients referred from 124 centres who underwent OTC at a central cryobank between 2000 and 2021.

**PARTICIPANTS/MATERIALS, SETTING, METHODS:**

Patients were stratified into children (<15 years, n = 149), adolescents (15–19 years, n = 296), and adults (≥20 years, n = 1,981). Residential addresses were geocoded, and distances to the cryobank were calculated. Regional distribution was categorised by population size. Statistical analyses included Chi-square and non-parametric tests with Bonferroni correction.

**MAIN RESULTS AND THE ROLE OF CHANCE:**

Median distance to the cryobank increased with age (children: 136 km; adolescents: 183 km; adults: 222 km), with children living significantly closer than adolescents (*p* = 0.03) and adults (*p* = 0.001). Overall, 44.8% of patients resided in large cities, with significant age-group differences (*p* < 0.001). Paediatric cases were concentrated in urban areas, whereas adults were more geographically dispersed. Among adults, distance varied by indication (*p* = 0.021), but not in younger groups. This pattern persisted despite the nationwide referral network.

**LIMITATIONS, REASONS FOR CAUTION:**

The retrospective design precludes causal inference. Distances were calculated as straight-line measures instead of real travel time, and socioeconomic factors were not available. Furthermore, only patients who successfully underwent OTC were available for analysis. Consequently, the study cannot assess true access to fertility preservation, referral rates, counselling rates, or the number of eligible patients who did not proceed to treatment. Although patients were referred from centres across Germany, cryopreservation was centralized in Bonn. Regional referral relationships and the structure of the referral network may therefore have influenced the observed geographic distribution, particularly in paediatric patients, and may limit generalisability to the entire German population.

**WIDER IMPLICATIONS OF THE FINDINGS:**

The findings demonstrate age-dependent geographic utilisation patterns among patients who underwent OTC. These patterns may reflect differences in referral pathways, counselling practices, and utilisation of fertility preservation services. However, because only patients who successfully underwent OTC were included, the study cannot directly assess referral rates, unmet need, or access barriers. Expanding structured referral pathways and reimbursement policies may improve equitable utilisation of fertility preservation services.

**FUNDING:**

Intramural funding from the University Hospital Bonn. This publication was supported by the Open Access Publication Fund of the University of Bonn. No external grant funding was received from any funding agency in the public, commercial, or not-for-profit sectors.

**DISCLOSURES:**

The authors declare that they have no competing interests.

**TRIAL REGISTRATION NUMBER:**

N/A

WHAT DOES THIS MEAN FOR PATIENTS? This study examined utilisation of ovarian tissue cryopreservation for girls and women with cancer. Ovarian tissue cryopreservation involves removing and freezing a small piece of an ovary before cancer treatment begins. The tissue may later be transplanted back to restore fertility and hormone function. For many young girls who have not yet reached puberty, this is currently the only established option to preserve the chance of having biological children in the future.

To better understand who receives this treatment, we analysed patients whose ovarian tissue was stored in one of Germany’s largest cryobanks, located in Bonn. We found that young children (under 15 years of age) who underwent ovarian tissue cryopreservation were more likely to live close to the cryobank. Children living farther away or in rural areas were less often represented than adolescents and adults.

These findings suggest that where a child lives and how they are referred to specialist fertility preservation services may influence whether they receive this treatment. This is important because every child who may benefit from fertility preservation should have the same opportunity to use it, regardless of where they live. Increasing awareness among healthcare professionals, improving referral pathways, and reducing practical or organisational barriers could help ensure more equal utilisation. Ovarian tissue cryopreservation itself is a well-established procedure; the challenge is making sure that all eligible children can benefit from it.

## Introduction

Ovarian tissue cryopreservation (OTC) has emerged as a key fertility preservation (FP) strategy for female cancer patients at risk of treatment-induced premature ovarian insufficiency (Jensen *et al.*, 2015; Gellert *et al.*, 2018; Yding Andersen *et al.*, 2019; Fraison *et al.*, 2023). In 2019, the American Society for Reproductive Medicine (ASRM) removed the experimental designation for OTC in postpubertal women and girls (Practice Committee of the American Society for Reproductive Medicine, 2019), reflecting substantial evidence from large single-centre cohorts demonstrating successful ovarian tissue transplantation (OTT) outcomes with live birth rates exceeding 30% in adults (Donnez *et al.*, 2015; Meirow *et al.*, 2016; Emrich *et al.*, 2025). For prepubertal girls, for whom oocyte or embryo cryopreservation is technically not feasible, OTC remains the only established FP option (Schallmoser *et al.*, 2023c), as confirmed by the PanCareLIFE Consortium (Mulder *et al.*, 2021).

Significant technological advances—including standardised tissue processing protocols, optimised slow-freezing and vitrification methods (Meirow *et al.*, 2015; Schallmoser *et al.*, 2023a, 2023b; Sänger *et al.*, 2024; Färber *et al.*, 2025), and rigorous quality control—have established OTC as a reliable clinical intervention. Robust clinical evidence, including individual patient data meta-analyses, demonstrates restoration of both reproductive and endocrine function following transplantation (Kristensen and Andersen, 2018) across a wide range of indications, including benign diseases (Khattak *et al.*, 2022; Santulli *et al.*, 2023).

Despite these advances, real-world implementation remains uneven, particularly in paediatric populations. Data from the Danish national FP programme indicate variable retransplantation patterns, with many patients opting for long-term storage of cryopreserved tissue, highlighting gaps in counselling and follow-up (Kristensen *et al.*, 2021). Previous work has also emphasised ongoing challenges in patient selection, multidisciplinary coordination, and integration of FP into complex healthcare systems (Macklon *et al.*, 2014; Macklon, 2020; Macklon and Fauser, 2020).

Equitable access to FP extends beyond clinical eligibility to encompass structural determinants including cryobank availability, established referral networks, and clinician awareness. These factors are particularly relevant for paediatric patients, who rely entirely on healthcare providers for timely counselling. In oncology, geographic distance is a well-recognised barrier associated with delayed diagnosis, reduced treatment adherence, and poorer outcomes. Multiple studies have demonstrated that increased travel distance correlates with more advanced disease at diagnosis and reduced receipt of guideline-concordant therapy amongst rural patients (Singh *et al.*, 2011; Massarweh *et al.*, 2014; Ambroggi *et al.*, 2015; Blake *et al.*, 2017; Garcia *et al.*, 2019; Ray *et al.*, 2020; Elattabi *et al.*, 2025).

Similar disparities are likely to affect FP services. Specialised cryobanking facilities are typically concentrated in urban academic centres, potentially disadvantaging patients from rural or remote regions. Comparable patterns have been observed in male FP, where sperm banking rates decline with increasing distance from referral centres (Fillon, 2023; Pelzman *et al.*, 2023), and in female patients, where the likelihood of receiving fertility-sparing treatment decreases with greater distance from oncofertility clinics (Goodman *et al.*, 2012; Kanbergs *et al.*, 2024).

In this nationwide retrospective study, we analysed 2426 patients referred from 124 centres across Germany who underwent OTC at the University Hospital Bonn cryobank between 2000 and 2021. Patients were stratified by age into children (<15 years), adolescents (15–19 years), and adults (≥20 years). We hypothesised that paediatric OTC cases would be disproportionately concentrated in geographically proximate urban regions, potentially reflecting differences in referral pathways and utilisation patterns across age groups.

## Materials and methods

### Study population and geographic distribution

This nationwide retrospective study included 2426 patients who underwent OTC at the University Hospital Bonn. Patients were referred from 124 centres located in 91 cities across Germany between 2000 and 2021, representing a nationwide multicentre referral cohort. The study included female children (0–14 years), adolescents (15–19 years), and adults (≥20 years) who underwent OTC. Age group definitions follow established classifications previously described (Arapaki *et al.*, 2022; Siegel *et al.*, 2023). All patients who underwent OTC at the University Hospital Bonn between 2000 and 2021 were included following provision of written informed consent. Participants received comprehensive fertility counselling and risk stratification per national guidelines. Inclusion criteria were female sex. Exclusion criteria included male sex, distant metastases, or a markedly reduced survival probability (Deutsche Gesellschaft für Gynäkologie und Geburtshilfe (DGGG), Deutsche Gesellschaft für Reproduktionsmedizin (DGRM), Deutsche Gesellschaft für Urologie, 2025). Data extraction included all eligible cases within this period. Of the 2475 patients who underwent OTC during the study period, residential address data were available for 2426 patients and regional classification data for 2470 patients; these subsets were included in the respective geospatial analyses. Until 2023, German patients bore full OTC and storage costs independently.

**Figure 1.**
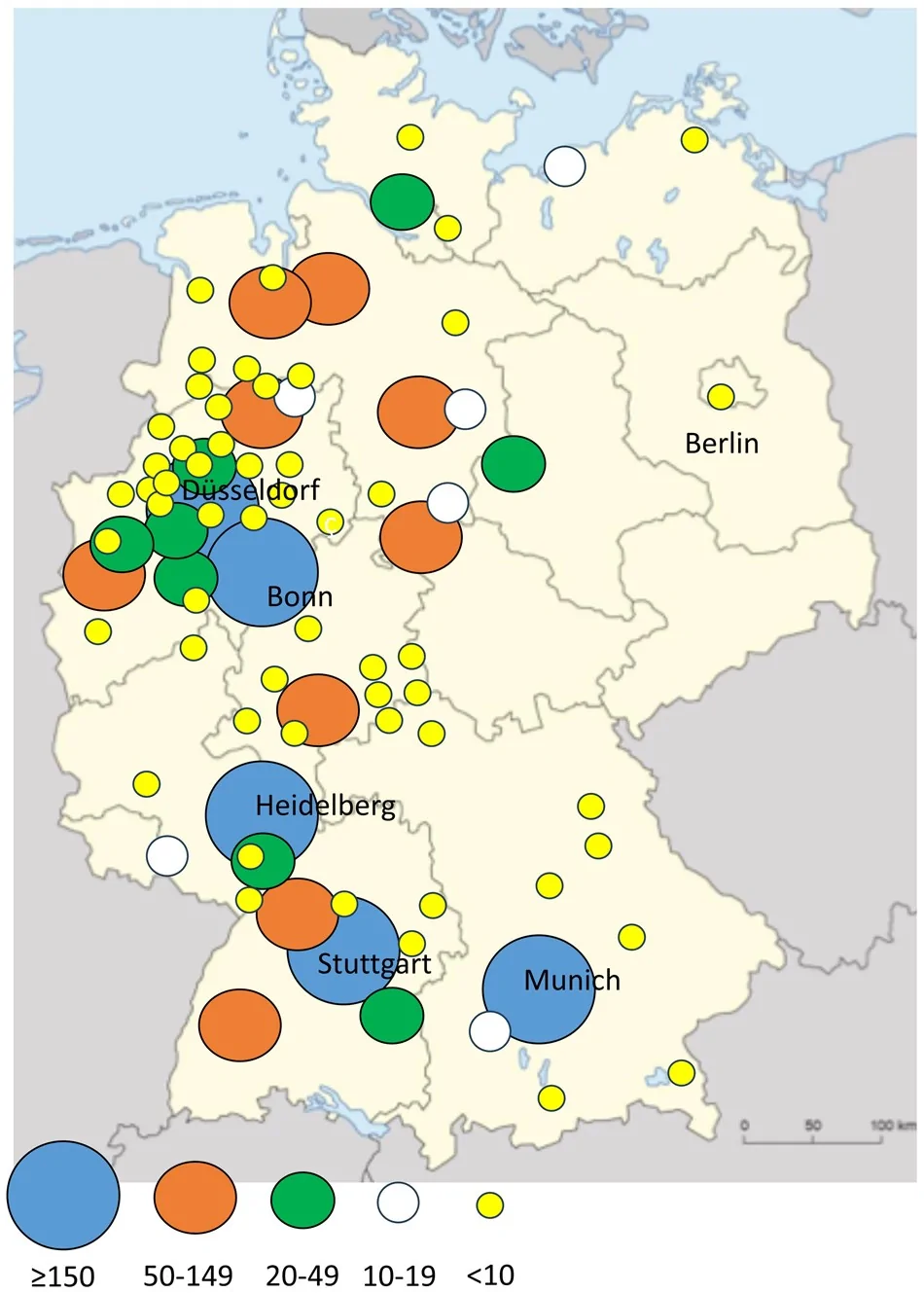
**Geographic distribution of referral centres performing ovarian tissue harvesting for ovarian tissue cryopreservation (OTC).** The colour and size of each circle correspond to the number of patients undergoing ovarian tissue collection at that centre.

In Germany, ovarian tissue obtained from external centres is predominantly transferred to three major cryobank sites—University Hospital Düsseldorf, University Hospital Erlangen, and University Hospital Bonn—for cryopreservation and long-term storage. Among these, the facility in Bonn is the largest. Although cryopreservation was centralised at the University Hospital Bonn, ovarian tissue was obtained from 124 referring centres across 91 cities, reflecting a nationwide multi-centre referral network rather than a single-centre population (see Fig. 1). Residential addresses were geocoded using BatchGeo (BatchGeo LLC, Chicago, IL, USA). Euclidean distances were subsequently calculated in Microsoft Excel (Microsoft Corporation, Redmond, WA, USA) using the geocoded latitude and longitude coordinates.

Straight-line (Euclidean) distance was selected as a pragmatic measure of geographic accessibility. Previous healthcare accessibility studies have demonstrated a very strong correlation between Euclidean distance and actual travel measures, including road travel distance and travel time, supporting its use in large population-based analyses where detailed transport data are unavailable (Phibbs and Luft, 1995; Apparicio *et al.*, 2008). Residential areas were classified into four predefined population-based categories: large cities (>100 000 inhabitants), medium-sized cities (20 000–100 000 inhabitants), small towns (5000–20 000 inhabitants), and rural communities (<5000 inhabitants).

### Patient data

Indications for OTC were classified into eight categories: haematological malignancies; brain and nervous system tumours; sarcomas; gynecological malignancies (epithelial ovarian carcinoma, germ cell tumours, granulosa cell tumours, borderline tumours, cervical squamous cell carcinoma, cervical adenocarcinoma, hydatidiform mole, choriocarcinoma, vulvar carcinoma); breast cancer; lymphomas; other conditions (Turner syndrome, galactosemia, sickle cell disease, thalassemia major, medullary aplasia, Kostmann syndrome); and unspecified diagnoses (when surgery occurred at external facilities without disease subtype documentation).

For malignancies associated with a high risk of malignant cell reintroduction following OTT, patients and their parents were counselled that *in vitro* growth (IVG) and *in vitro* maturation (IVM) – currently experimental techniques—represent the only potential utilisation options for the cryopreserved tissue.

### Statistical analysis

Statistical analyses included descriptive statistics, Chi-square tests for categorical variables, and Kruskal–Wallis tests for continuous variables. When overall group differences were significant, post hoc pairwise comparisons were performed using Mann–Whitney *U* tests with Bonferroni correction. Effect sizes were calculated and reported as Cohen’s *d*. All analyses were carried out using IBM SPSS Statistics for Windows, Version 31.0 (IBM Corp., Chicago, IL, USA), and statistical significance was set at *P* < 0.05. Given the exploratory and predominantly descriptive nature of the study, analyses were designed to identify geographic patterns and differences between age groups rather than to estimate causal effects.

### Tissue procedures

Ovarian tissue harvesting was performed laparoscopically at the University Hospital Bonn or at referring centres, typically as a standalone procedure. In children, one entire ovary was preferentially removed to ensure sufficient tissue for cryopreservation. In adults and postpubertal adolescents, approximately 50% of one ovary was excised (Deutsche Gesellschaft für Gynäkologie und Geburtshilfe (DGGG), Deutsche Gesellschaft für Reproduktionsmedizin (DGRM), Deutsche Gesellschaft für Urologie, 2025). Immediately post-resection, tissue was preserved in Custodiol medium (Dr. Köhler, Bensheim, Germany) at 4°C. A separate fragment was formalin-fixed for histological metastasis screening.

Transport of ovarian tissue to the centralised cryobank in Bonn was performed using custom transport containers containing Custodiol medium, cold packs, temperature loggers (delta T, Fernwald, Germany), and insulating inlays (Consarctic, Westerngrund, Germany). Delivery occurred via overnight shipment or same-day transport (Schallmoser *et al.*, 2023c).

Tissue processing followed previously published protocols by Schallmoser *et al.* (2022, 2023a): medulla ablation, sterile cortical strips cut to 8 × 4 × 1 mm (reduced to 5 × 4 × 1 mm from 2019), collagenase viability staining, then cryopreservation via slow-freezing (Rivas Leonel *et al.*, 2019) or vitrification (Schallmoser *et al.*, 2023a, 2023b; Sänger *et al.*, 2024) preserving metabolic activity, follicular integrity, and angiogenic signalling after thawing (Einenkel *et al.*, 2022, 2024). Storage occurred in vapour-phase liquid nitrogen (−160°C) within automated, alarmed 300-L tanks (MVE/Chart Biomedical, Ball Ground, GA, USA; Planer plc, Sunbury-on-Thames, UK).

### Ethical approval

The study protocol and use of clinical cryobank data were approved by the Ethics Committee of the University Hospital Bonn (approval code 007/09) in accordance with the Declaration of Helsinki. Informed consent was obtained as applicable. For minor patients, written cryopreservation consent was obtained from parents or legal guardians. Upon reaching adulthood, children and adolescents were required to re-execute their cryopreservation consent agreements.

## Results

Analyses were conducted on two subsets: distance analyses (n = 2426 with available address data) and regional analyses (n = 2470 with regional classification data). Of the 2426 patients who underwent OTC between 2000 and 2021, 149 (6.1%) were children (<15 years), 296 (12.2%) adolescents (15–19 years), and 1981 (81.7%) adults (≥20 years).

### Residential distances

**Figure 2.**
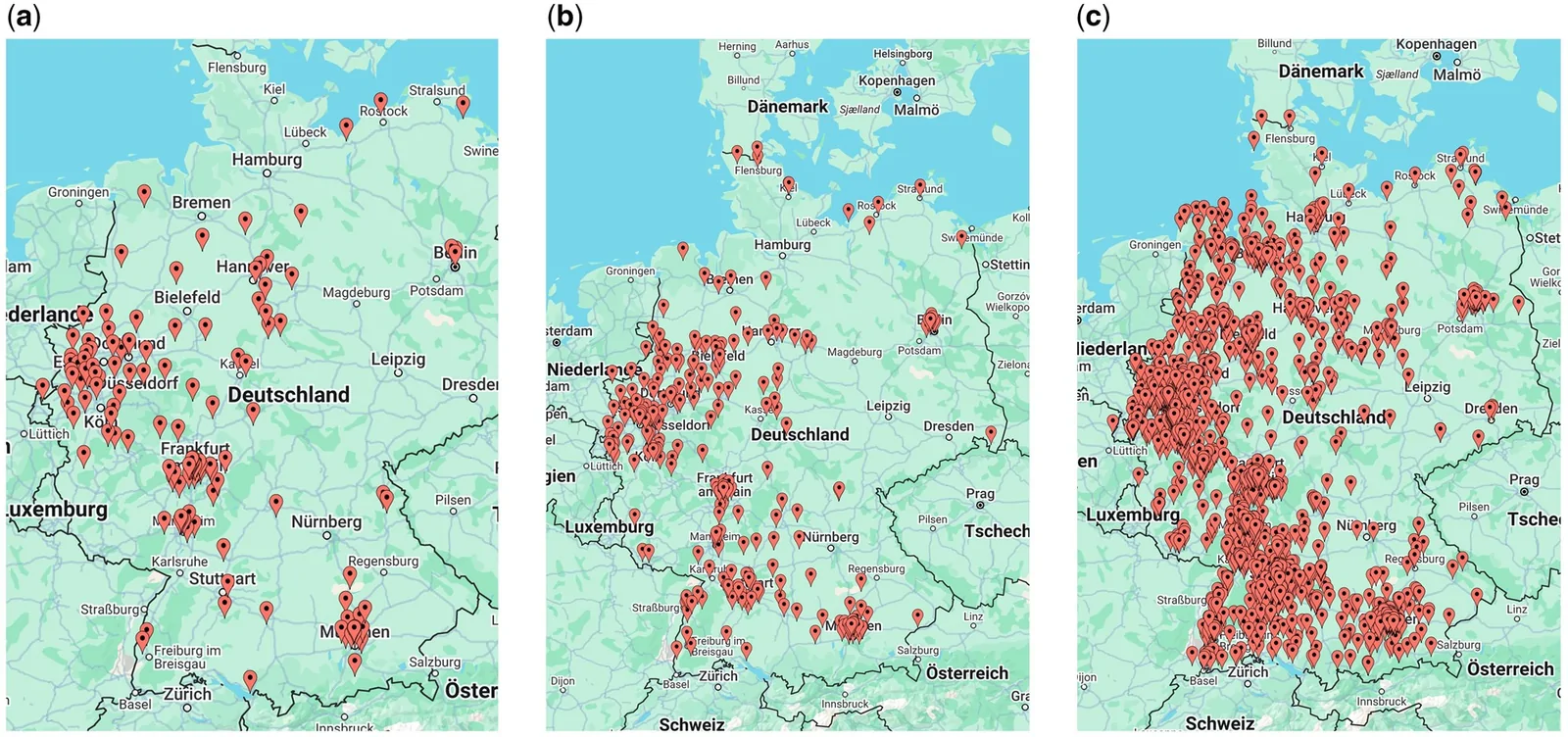
**Geographic distribution of the residential locations of patients undergoing ovarian tissue cryopreservation (OTC), by age group.** Maps display residential locations of (**a**) children (<15 years), (**b**) adolescents (15–19 years), and (**c**) adults (≥20 years) at the time of OTC, illustrating increasing spatial dispersion with advancing age.

**Figure 3.**
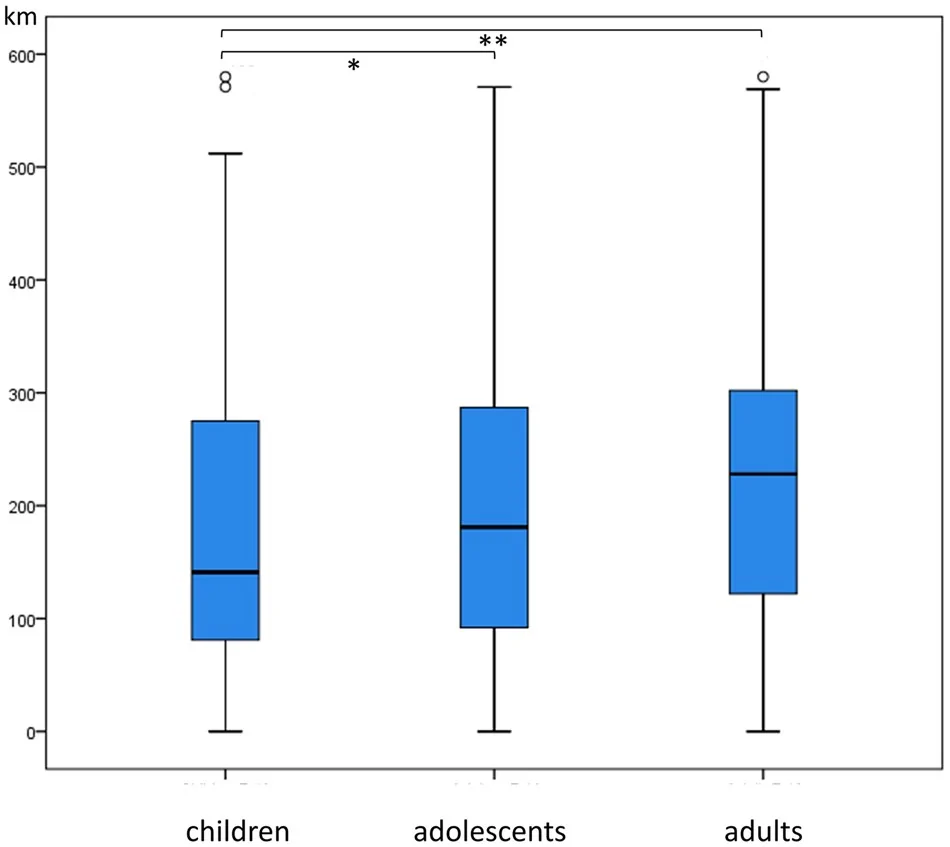
**Median residential distance from the patients’ place of residence to the University Hospital Bonn cryobank, by age group.** Box plots display median (central line), interquartile range (box), and full range (whiskers) for children (<15 years, n = 149; median 136 km), adolescents (15–19 years, n = 296; median 183 km), and adults (≥20 years, n = 1,981; median 222 km). Overall group differences were analysed using the Kruskal–Wallis test. When the overall test was significant, post hoc pairwise comparisons were performed using Mann–Whitney *U* tests with Bonferroni correction. *Indicates children versus adolescents (*P* = 0.03); **indicates children versus adults (*P* = 0.001).

Median residential distances to the cryobank were 136 km (mean 187.1 km, standard deviation (SD) ± 140.8 km, 95% confidence interval (95% CI) 164.9–209.3) for children, 183 km (mean 210.2 km, ±SD 137.6 km, 95% CI 194.7–225.7) for adolescents, and 222 km (mean 223.1 km, SD ±140.1 km, 95% CI 217.0–229.3) for adults. Distances differed significantly (*P* = 0.0017). Post hoc analysis showed that children lived closer than adolescents (*P* < 0.05, Cohen’s *d* = 0.2) and adults (*P* < 0.001, Cohen’s *d* = 0.3), whereas no significant difference was observed between adolescents and adults (*P* = 0.106) (Figs 2 and 3; Table 1).

**Table 1.** **Residential distance from the patients’ place of residence to the** University Hospital Bonn **cryobank according to age group.**

Age group	n	Median (km)	IQR	Min–max
Children	149	**136.0** * ^,^ ^†^	193	2–580
Adolescents	296	**183.0** *	191	0–571
Adults	1981	**222.0** ^†^	219	0–580

IQR, interquartile range. Overall group differences were analysed using the Kruskal–Wallis test. When the overall test was significant, post hoc pairwise comparisons were performed using Mann–Whitney *U* tests with Bonferroni correction.Bold median values indicate age groups involved in at least one statistically significant pairwise comparison; identical symbols denote the corresponding comparison:

* Indicates children versus adolescents (*P* < 0.05).

† Indicates children versus adults (*P* < 0.001).

### Patient distribution by region

Overall, 44.8% of patients (1106/2469) resided in large cities (≥100 000 inhabitants), with representation decreasing across less urban regions (Table 2). Significant age-group differences were observed in large (*P* < 0.001) and medium-sized cities (20 000–100 000 inhabitants; *P* < 0.001), whereas distributions in small towns (5000–20 000 inhabitants) and rural areas (<5000 inhabitants) were similar across age groups (23.5–21.2% and 7.4–9.3%, respectively) (Table 2). Bonferroni-corrected post hoc analysis identified significant pairwise differences. In large cities, adolescents were underrepresented compared with adults (30.7% vs. 47.1%, *P* < 0.001). In medium-sized cities, children were significantly less frequently represented than adolescents (26.8% vs. 38.7%, *P* < 0.05), whereas adolescents were significantly more frequently represented than adults (38.7% vs. 25.0%, *P* < 0.001). Children showed the highest concentration in large cities (42.3%, 63/149), with significantly lower representation in medium-sized cities compared with adolescents and decreasing representation across less urban regions (Table 2).

**Table 2.** Distribution of patients according to residential region and age group.

Region	Children (n = 149)	Adolescents (n = 302)	Adults (n = 2,019)	Total (n = 2,470)
Large city (≥100k)	63 (42.3%)	**93 (30.7%)** *	**951 (47.1%)** *	1,107 (44.8%)
Medium city (20–100k)	**40 (26.8%)** **	**117 (38.7%)** ** ^,^ ^†^	**505 (25.0%)** ^†^	662 (26.8%)
Small town (5–20k)	35 (23.5%)	64 (21.2%)	394 (19.5%)	493 (20.0%)
Rural (<5k)	11 (7.4%)	28 (9.3%)	169 (8.4%)	208 (8.4%)

Residential regions were classified as follows: large city (>100 000 inhabitants), medium-sized city (20 000–100 000 inhabitants), small town (5000–20 000 inhabitants), and rural community (<5000 inhabitants). Overall group differences were analysed using the Chi-square test. When the overall test was significant, *post hoc* pairwise comparisons with Bonferroni correction were performed. Bold values indicate age groups with a statistically significant pairwise difference within the respective residential-region category; identical symbols denote the corresponding comparison:

* Indicates adolescents versus adults for large cities (*P* < 0.001).

** Indicates children versus adolescents for medium-sized cities (*P* < 0.05).

† Indicates adolescents versus adults for medium-sized cities (*P* < 0.001).

### Distance by cancer type

Among adults, residential distance to the cryobank varied by cancer indication (*P* = 0.021, Cohen’s *d* = 0.3). Post hoc analysis showed shorter mean distances for non-specified indications (185.3 km, SD ± 146.6, 95% CI 159.5–211.2) compared with lymphoma (233.5 km, SD ± 137.9, 95% CI 219.9–247.1) (Table 3). No indication-specific differences were observed in children or adolescents. For example, median distance was 113.0 km for paediatric gynaecological tumours (n = 7) and 251.2 km for adolescent sarcomas (n = 47) (Table 3).

**Table 3.** Residential distance from the patients’ place of residence to the University Hospital Bonn cryobank according to cancer type for children, adolescents, and adults.

Cancer type	Children			Adolescents			Adults		
	n	Mean ± SD (km)	95% CI	n	Mean ± SD (km)	95% CI	n	Mean ± SD (km)	95% CI
Haematological malignancies	12	165.8 ± 134.9	80.1–251.6	17	124.9 ± 83.7	81.9–167.9	31	240.4 ± 162.3	180.9–299.9
Brain and nervous system tumours	11	200.5 ± 146.4	102.1–299.0	15	240.3 ± 149.1	157.7–322.9	45	250.9 ± 139.3	209.0–292.7
Sarcoma	37	225.1 ± 152.7	174.2–276.0	47	251.2 ± 155.1	205.6–296.7	57	232.6 ± 119.2	201.0–264.2
Gynaecological tumours	7	113.0 ± 113.6	8.0–218.2	26	196.4 ± 142.5	138.8–254.0	123	226.4 ± 156.7	198.4–254.4
Breast cancer	0	–	–	4	199.0 ± 120.1	7.8–390.2	1,085	220.7 ± 137.9	212.5–229.0
Lymphoma	23	190.4 ± 169.1	117.2–263.5	126	215.4 ± 133.9	191.8–239.1	398	**233.5 ± 137.9** **	219.9–247.1
Other conditions*	49	188.3 ± 120.9	153.6–223.0	38	201.0 ± 119.7	161.6–240.3	116	229.1 ± 139.8	203.4–254.8
Non-specified^†^	10	113.7 ± 76.5	59.0–168.4	23	188.0 ± 141.5	125.3–250.7	126	**185.3 ± 146.6** **	159.5–211.2

* Other conditions include Turner syndrome, galactosemia, sickle cell disease, thalassemia major, medullary aplasia, and Kostmann syndrome.

† Non-specified indicates cases in which ovarian tissue harvesting was performed at an external facility without documentation of the disease subtype. SD, standard deviation; CI, confidence interval. Overall group differences were analysed using the Kruskal–Wallis test. When the overall test was significant, post hoc pairwise comparisons were performed using Mann–Whitney *U* tests with Bonferroni correction.

Bold mean ± SD values marked with ** indicate a significant difference between lymphoma and non-specified cases in adults (*P* = 0.021).

### Temporal sensitivity analysis

To evaluate temporal changes in fertility preservation practice, referral structures, reimbursement policies, and clinical awareness, a sensitivity analysis was performed across three treatment periods (2000–2009, 2010–2015, and 2016–2021). During 2000–2009, no significant difference in residential distance between paediatric and adult patients was observed. In this period, OTC was performed in 10 children. During 2010–2015, paediatric patients lived significantly closer to the Bonn cryobank than adults (median 131.0 km, mean 185.5 km, SD ±128.1, 95% CI 145.6–225.4 vs. median 230.0 km, mean 229.2 km, SD ±137.2, 95% CI 220.4–238.1; *P* < 0.05, Cohen’s d = 0.3). A total of 42 children underwent OTC during this period. Similarly, during 2016–2021, paediatric patients lived significantly closer to the Bonn cryobank than adults (median 141.0 km, mean 194.5 km, SD ±145.8, 95% CI 165.1–223.8 vs. median 224.5 km, mean 225.3 km, SD ±144.7, 95% CI 214.9–235.8; *P* < 0.05, Cohen’s *d* = 0.2). During this period, 97 children underwent OTC. No significant differences in residential distance were observed between children and adolescents or between adolescents and adults in any of the three time periods.

## Discussion

This nationwide multicentre referral study demonstrates marked age-dependent geographic differences among patients who underwent OTC. Paediatric patients were predominantly located in the local Bonn region and in urban areas, whereas adolescents and adults were referred from more distant regions. Because only patients who successfully underwent OTC were included, the present study cannot determine true referral rates, unmet need, or access barriers. The observed geographic patterns may reflect differences in referral pathways, counselling practices, and utilisation of fertility preservation services across age groups. These mechanisms may include structural drivers, such as the centralisation of paediatric oncology and reproductive medicine services, and behavioural drivers, such as clinician awareness and referral practices. Sensitivity analysis stratified by treatment era revealed no significant differences between paediatric and adult patients during 2000–2009, likely reflecting the small number of paediatric cases during this period, when OTC was performed less frequently in children (Emrich *et al.*, 2025). In contrast, during both subsequent periods (2010–2015 and 2016–2021), paediatric patients lived significantly closer to the cryobank than adults. These findings indicate that the observed age-dependent geographic pattern was consistent across different eras of clinical practice.

In paediatric patients, fertility preservation counselling and referral are largely clinician-initiated, which may contribute to the observed geographic clustering near the cryobank. In addition, the centralisation of paediatric oncology care within specialised tertiary centres may contribute to the observed geographic patterns independently of referral behaviour alone.

A cancer diagnosis is often prioritised over future fertility considerations, potentially limiting engagement with fertility preservation despite evidence that fertility becomes an important concern for many survivors, with 76% expressing a desire for parenthood after remission (Schover *et al.*, 1999). Early and structured counselling is therefore essential. Fertility preservation counselling remains less frequent in paediatric patients, possibly reflecting the greater temporal distance to parenthood and a greater reliance on clinician-initiated discussions. Although counselling rates increased from 33% in the early 1980s to approximately 50% in the early 2000s (Hohmann *et al.*, 2011), these findings underscore the need to better integrate fertility preservation counselling into routine oncological care, particularly for paediatric patients.

### Possible structural reasons for geospatial differences

In Germany, reproductive medicine services at university hospitals have declined in recent years. Currently, only 19 of 38 university hospitals maintain reproductive medicine departments, and six federal states lack university-based reproductive medicine services altogether (Deutsche Gesellschaft für Gynäkologie und Geburtshilfe e.V., 2023). As ovarian tissue harvesting is primarily performed at university hospitals, this limited infrastructure may contribute to regional gaps in access to fertility preservation services. OTC in Germany has historically been organised through a limited number of specialised cryobanks. Erlangen represents one of the earliest established ovarian tissue cryopreservation programmes in Germany, with published data including patients who underwent ovarian tissue harvesting and cryopreservation since 1998 (Lotz *et al.*, 2016). Bonn and Erlangen subsequently developed into the two major long-standing cryobanks within the German fertility preservation network. By July 2015, approximately 1400 tissue samples had been stored in Bonn compared with approximately 500 in Erlangen (van der Ven *et al.*, 2016). A subsequent analysis from the Bonn cryobank reported ovarian tissue cryopreservation in 2475 patients between 2000 and 2021, reflecting its prominent role within the FertiPROTEKT network (Schallmoser *et al.*, 2023c). Additional cryopreservation capacity became available later in other centres, including Düsseldorf, where published ovarian tissue cryopreservation activity has been reported from 2018 onwards (Baston-Büst and Bielfeld, 2022). The prominent role of the Bonn cryobank within the German fertility preservation network may have influenced referral patterns and the geographic distribution of patients undergoing ovarian tissue cryopreservation.

Alternative explanations should also be considered. The observed geographic differences may reflect established regional referral networks, whereby patients are preferentially referred within existing collaborations between oncology centres, reproductive medicine units, and cryobanks. In addition, the centralisation of paediatric oncology care may influence referral patterns, as specialised treatment and fertility preservation services are often concentrated in tertiary centres. Because data on proximity to paediatric oncology centres, reproductive medicine units, and local healthcare resources were not available, the present study cannot determine the extent to which these factors contributed to the observed geographic patterns. Further studies including referral pathways and healthcare infrastructure data are needed to clarify these factors.

### Possible behavioural reasons for geospatial differences

Clinician awareness of paediatric OTC is likely higher in tertiary children’s hospitals, where multidisciplinary tumour boards routinely assess fertility risks as part of treatment planning and facilitate timely referral for fertility preservation. These centres are also more likely to incorporate established fertility preservation guidelines from ASCO (Su *et al.*, 2025) or ISFP (Kim *et al.*, 2012) into clinical practice. In contrast, smaller paediatric oncology departments may encounter eligible patients less frequently and have less structured fertility preservation pathways, potentially resulting in lower awareness and less consistent counselling and referral practices.

OTC in children is more complex than in adults and requires multidisciplinary coordination involving paediatric surgeons, reproductive specialists, and oncologists. Harvesting ovarian tissue, particularly in very young patients, requires specialised surgical expertise, frequently with reproductive specialists assisting at the operating table. Such coordination is typically feasible only in specialised tertiary centres. Facilitating access to specialised centres is therefore essential to improve equitable access for paediatric patients.

Urban–rural differences in OTC utilisation may partly reflect socioeconomic factors, as German central regions show 7–20% higher average incomes than peripheral/rural areas, potentially mediating awareness and utilisation differences (Institute for Employment Research, 2022). Until 2023, German patients bore full OTC and cryopreservation costs (approximately €3,000–5,000 upfront + €200 − 400/year storage). Currently, coverage applies only to postmenarcheal girls (Gemeinsamer Bundesausschluss, 2022). Information on socioeconomic status and healthcare infrastructure was not available in this study and therefore could not be included in the analyses.

### Impact of travel burden in different patient groups

Long travel distances to healthcare facilities have been associated with delayed diagnoses, reduced adherence to guideline-concordant treatment, and poorer outcomes in oncology, particularly among rural populations (Singh *et al.*, 2011; Massarweh *et al.*, 2014; Ambroggi *et al.*, 2015; Blake *et al.*, 2017; Garcia *et al.*, 2019; Ray *et al.*, 2020; Elattabi *et al.*, 2025).

Similar barriers have been reported in fertility preservation, where increasing distance has been associated with lower rates of semen cryopreservation and fertility-sparing treatment (Fillon, 2023; Pelzman *et al.*, 2023; Kanbergs *et al.*, 2024), although findings are not entirely consistent and may depend on distance thresholds (Goodman *et al.*, 2012). Access challenges remain relevant, with 5.7% of reproductive-age women in the United States living more than two hours from an oncofertility clinic (Peipert *et al.*, 2023). In Germany, specialised services such as cryobanks are concentrated in urban centres despite 57% of the population living in rural areas (Thünen-Institut, 2026). These findings are consistent with our results and highlight the need for regional care models that combine centralised expertise with effective referral and outreach networks.

### Indication patterns for cryopreservation

Indication patterns were consistent with previous reports (Schallmoser *et al.*, 2023c; Emrich *et al.*, 2025). In paediatric patients, residential distance to the cryobank did not differ by indication, suggesting relatively uniform fertility preservation counselling and referral across tumour entities. In adults, distance patterns were more heterogeneous, with lymphoma patients tending to live farther away than patients with non-specified indications, likely reflecting referrals from nearby collaborating centres with incomplete documentation. These findings suggest that, in addition to geography, clinician engagement and documentation practices may influence referral patterns and data quality, highlighting the need for standardised referral pathways and indication reporting.

### Solution approaches

Current guidelines recommend early fertility preservation counselling in paediatric oncology and recognise OTC as a standard option for appropriate candidates (Practice Committee of the American Society for Reproductive Medicine, 2019; Deutsche Gesellschaft für Gynäkologie und Geburtshilfe (DGGG), Deutsche Gesellschaft für Reproduktionsmedizin (DGRM), Deutsche Gesellschaft für Urologie, 2025). Consistent implementation through clinician education, structured referral pathways, and coverage of costs for prepubertal patients may help reduce the geographic disparities observed in this study.

Improving clinician education is a key strategy to enhance FP awareness, since FP counselling rates increased from 6.7% before targeted education to 46% afterward (Schover *et al.*, 1999). Sustained improvements likely require regular training integrated into oncology curricula to standardise FP counselling practice.

Expanding referral networks is a key strategy, as ovarian tissue can be safely transported for up to 21 h post-harvest without loss of viability (Erden *et al.*, 2026), enabling regional collaboration between harvesting centres and specialised cryopreservation facilities to overcome geographic barriers (Kristensen *et al.*, 2021; Schallmoser *et al.*, 2026). In Germany, however, TPG (transplant law) and AMG (medicines act) regulations governing tissue handling and transport add complexity and may hinder collaboration between hospitals and cryobanks (Gesundheitswesen, 2026), underscoring the need for specialised centres to support partner sites in regulatory processes. In addition, online decision aids for patients and parents may complement counselling; such tools have been shown to facilitate earlier FP decisions (Ehrbar *et al.*, 2019). While not a substitute for specialist consultation, they can improve awareness of options and support timely decision-making, particularly in underserved regions.

Since urban−rural wage gaps likely contribute to differences in utilisation, cost structures could represent an important determinant of utilisation. Limited public reimbursement further disadvantages self-paying patients, amplifying inequities driven by socioeconomic status. While recent policy changes enabling insurance coverage for postpubertal OTC show feasibility, financial aid for prepubertal OTC remains limited. Targeted subsidies or transport grants could help reduce socioeconomic and geographic disparities in uptake, with broader international relevance for equitable oncofertility care.

From a systems perspective, national programmes demonstrate how coordinated care improves FP access and utilisation. The Danish FP programme achieved high uptake and substantial long-term storage rates, highlighting the importance of structured counselling, follow-up, and integrated infrastructure (Bach *et al.*, 2020; Kristensen *et al.*, 2021; Yde *et al.*, 2025a, 2025b). Broader reproductive medicine literature further emphasises the need for context-based approaches that address inequities in access, standardised pathways, and resource allocation across diverse patient populations and real-world settings (Macklon, 2020; Macklon and Fauser, 2020). Dittrich *et al.* emphasise that prompt and timely collaboration with specialised centres is essential (Dittrich and Lotz, 2023).

### Strengths and limitations

#### Strengths

This study comprises the largest cohort of patients undergoing OTC (n = 2426), referred from 124 centres across 91 cities in Germany. The dataset spans two decades with consistent cryopreservation protocols and includes comprehensive geocoded residential data and predefined regional classifications, enabling robust geospatial analyses.

#### Limitations

Euclidean (‘as-the-crow-flies’) distances systematically underestimate actual travel burden compared with road travel distance and travel time, particularly in rural areas with poor infrastructure. However, previous healthcare accessibility studies have reported very strong correlations between Euclidean distance and network-based travel measures, often exceeding correlation coefficients of 0.95 (Phibbs and Luft, 1995; Apparicio *et al.*, 2008), supporting its use as a proxy for geographic accessibility in large population-based studies. Nevertheless, the degree of underestimation may vary across geographic regions and local transport networks.

Lack of socioeconomic status, insurance coverage, or family income data prevents disentangling urban proximity from economic determinants of utilisation. In addition, data regarding proximity to specialised paediatric oncology centres, reproductive medicine units, and regional healthcare infrastructure were unavailable. Consequently, potential confounding by healthcare service availability and referral opportunities could not be evaluated. Regional classifications based on population size may mask intra-urban socioeconomic gradients or inter-regional healthcare infrastructure variations. Although multiple referral centres across Germany contributed to the cryobank at the University Hospital Bonn, the single-centre design limits the generalizability of the findings beyond Bonn’s catchment area, where regional predominance and integration within the FertiPROTEKT network may have increased external referrals. Furthermore, referral structures may differ between cryobanks according to regional collaborations, institutional expertise, and historical network development. Consequently, the geographic distribution observed in the present cohort should be interpreted as reflecting utilisation patterns within a large national referral network rather than as a direct representation of the German population.

As the 22-year study period covered a phase of considerable evolution in OTC practice, including changes in awareness, clinical procedures, and reimbursement policies, temporal bias cannot be excluded. However, stratified sensitivity analyses demonstrated that the principal findings remained consistent across the later treatment eras. Temporal address data reflect residence at cryopreservation, not diagnosis/transport origin, potentially underestimating barriers for patients. Multicentre studies incorporating travel-time modelling, socioeconomic status confounders, and longitudinal outcomes are warranted to validate findings nationally.

## Conclusions

This nationwide analysis identifies marked age-stratified geographic utilisation patterns among patients who underwent OTC. Paediatric cases were disproportionately clustered in urban areas and in close proximity to the cryobank at the University Hospital Bonn, in contrast to the broader geographic distribution observed in adults.

These findings may reflect differences in referral pathways, clinician awareness, healthcare infrastructure, and utilisation of fertility preservation services. However, residual confounding by socioeconomic and healthcare system factors cannot be excluded.

Standardised fertility preservation counselling, clinician education, and expanded tissue transport logistics could offer immediate solutions. Policy reforms for prepubertal OTC reimbursement and simplified regulations could enhance equity across urban–rural divides.

Future multicentre studies integrating travel-time analyses and socioeconomic factors are needed to validate these geographic utilisation patterns and evaluate intervention efficacy.

## Data Availability

The datasets used and/or analysed during the current study are available from the corresponding author on reasonable request.
